# Evaluation of the aquaporin molecules characterization in the sperm cells of men from different aged

**DOI:** 10.55730/1300-0144.5781

**Published:** 2024-01-05

**Authors:** Fikret GEVREK, Osman Serden GENCEL, Selim GÖRGÜN, Mikail KARA, Muzaffer KATAR

**Affiliations:** 1 Department of Histology and Embryology, Faculty of Medicine, Tokat Gaziosmanpaşa University, Tokat, Turkiye; 2 Department of Microbiology and Clinical Microbiology, Training and Research State Hospital, Samsun, Turkiye; 3 Department of Histology and Embryology, Faculty of Medicine, Hatay Mustafa Kemal University, Hatay, Turkiye; 4 Department of Biochemistry, Faculty of Medicine, Tokat Gaziosmanpaşa University, Tokat, Turkiye

**Keywords:** Aquaporin molecules, fertility, immunostaining, infertility, sperm

## Abstract

**Background/aim:**

Male infertility rises for many reasons, along with age; therefore, we aimed to research the characterization of aquaporin-3, 7, and 8 in human sperm belonging to different age groups.

**Material and methods:**

This study was conducted on sperm samples of men aged over 18 years. A total of 60 men were included in the study and divided into three age groups: group 1, age 18–25 years (n = 20); group 2, age 26–35 years (n = 20); and group 3, age ≥35 years (n = 20). Sperm ejaculates obtained from each participant were used for spermiogram tests, Kruger strict morphology analysis, and immunohistochemistry.

**Results:**

We observed no statistically significant differences in terms of macroscopic and microscopic sperm testing. The immunostaining score of aquaporin-3 was the lowest in group 1 and increased in group 3 and group 2, respectively (p < 0.05). Aquaporin-8 immunostaining only increased in group 2 (p < 0.05). Aquaporin-7 immunostaining scores were not different between the groups (p > 0.05). When the immunostaining scores of aquaporin molecules were compared with each other, aquaporin-7 was significantly increased compared with the others (p < 0.05).

**Conclusion:**

According to the results, it can be stated that aquaporin-3 and aquaporin-8 molecules were more expressed at age 26 to 35 years, and aquaporin-7 was densely expressed from age 18 to 25 years. If the characterization of these molecules is adversely affected, male infertility may eventually emerge. We recommend further advanced-level studies on this subject.

## 1. Introduction

Male infertility is considered as the inability of a man to satisfy his reproductive aims through regular unprotected sexual intercourse, which accounts for about 30% of infertility cases [1–3]. According to recent World Health Organization (WHO) statistics, nearly 50–80 million people across the world experience infertility [2, 4]. Some cases result from known and specific reasons such as chromosomal anomalies, infections, gene mutations, varicocele, hormonal disorders, or reproductive tract obstructions [5]. These can be temporary or permanent, and can also lead to some sperm abnormalities related to density, motility, morphology, and viability.

The public effect of infertility can be regarded as high because nearly 15% of couples of reproductive ages are unable to have a child after one year of unprotected intercourse [5]. This situation has recently become an increasingly important public health matter [6, 7].

Achieving fertility requires the normal functioning of both sexual reproductive system organs, structurally and functionally. Reproductive impairments can occur in either partner in a couple or both of them. It has been shown that approximately half of all infertility cases occur owing to female factors, 20%–30% to male factors, and 20%–30% due to common reasons of both sexes [2, 4]. In the most recent meta-analysis studies, it has been shown that male factors are present in 20%–70% of infertility cases [8]. These literature observations imply that male infertility cases are notably increasing steadily across the globe with time. Therefore, it is crucial to research male factors that can cause male infertility.

Despite all diagnostic advancements, in some infertility cases, the cause remains unexplained and these patients do not respond to treatments. This is called idiopathic infertility, constituting 30% of infertile men according to the 2015 guide of the European Association of Urology (EAU) [1, 9].

Spermatozoa proteins play an important role in sperm morphology, motility, vitality, and all physiologic events that enable sperm to achieve fertilization [10]. Some idiopathic infertility cases may occur due to several reasons associated with protein molecules expressed in sperm cells. In this sense, it has been suggested that male infertility may occur owing to the impairment of some sperm protein molecule expression related to genetic malfunctions [11]. According to both the EAU and the American Association of Urology (AAU) guidelines, genetic analysis is required in some infertility cases because chromosome anomalies are frequently seen [11].

Aquaporins (AQPs) are sperm proteins that are highly preserved and specialized integral plasma membrane proteins that facilitate water movement across the cell membrane [12,13]. AQPs were first discovered by Agre et al. in 1992 and 13 distinct isoforms from AQP-0 to AQP-12 have been identified since their discovery [14,15]. AQPs play a particularly important role in healthy and component sperm cells in production because they have an active role in the regulation of water and ion homeostasis in all biological organisms [16]. Some isoforms of AQPs have also been identified in sperm cells [17, 18]. Dysfunction of and/or the expression pattern of AQPs might be related to the occurrence of male infertility.

Many reasons such as chronic diseases, adverse environmental factors, drugs, and chemical agents, age, long-term exposure to electromagnetic fields emitted from mobile phones, and/or other sources may also cause male infertility through oxidative stress effects [16, 17]. If these irritating conditions steadily continue, the expression pattern and functions of AQPs may be affected negatively and male infertility can eventually appear.

As mentioned above, AQPs are very important for sperm health and function and their malfunction may be linked with male infertility. In the literature, the available data are scant and inconsistent and few studies directly handle the expression pattern of AQPs in male infertility. Therefore, in this study, we aimed to investigate AQP-3, 7, and 8 molecule characterization in human sperm cells of different ages to contribute to the literature related to the research of AQP molecule expression in human sperm.

## 2. Materials and methods

### 2.1. Experimental design

Before conduct of the research, written informed consent was taken from all participants, and approvals of both Tokat Gaziosmanpaşa University Human Experiments and Clinical Research Ethics Committee (18-KAEK-093) and Samsun Training and Research Hospital Ethics Committee (TUEK 191-2018 GOKAEK/13-96) were obtained because the study was planned in two centers. The procedures followed were in accordance with the ethical standards of the responsible organization and with the Helsinki Declaration of 1975 as revised in 1983.

For semen samples, a total of 60 men who applied for infertility aged over 18 years with 2–7 days of sexual abstinence were included in the study. However, those who were addicted to substances such as alcohol or drugs, those who had an active illness, those who took medication or hormone treatment, and those who complained of varicocele were not included in the study. The men were divided into three different age groups as follows: Group 1, age 18 to 25 years (n = 20); Group 2, age 26 to 35 years (n = 20); and Group 3, age ≥ 36 years (n = 20). The men gave sperm samples into sterile urinary dishes through masturbation. After the collection of semen samples, sperm smear preparations, fixation of the sperm smears with 4% buffered neutral formalin, and spermiogram analysis were performed in Samsun Training and Research Hospital Andrology Laboratory. Spermiogram tests were conducted using a spermiogram analyzer (MES-Medical Electronic Systems, SQA-V Gold, Israel). Formalin-fixed smear slides stayed at 4 °C in the refrigerator. On the last day of semen collection, the slides were transferred to the laboratory of the Department of Histology and Embryology at Tokat Gaziosmanpaşa University within the same day by OSG (study member) with his car.

The Kruger strict sperm morphology analyses, immunohistochemical staining, and microscopic analyses of the study were performed in the laboratories of the Department of Histology and Embryology at Tokat Gaziosmanpaşa University. The analysis of the sperm samples was conducted by a researcher who was blinded to the groups.

### 2.2. Spermiogram analysis

Spermiogram tests were performed based on the WHO 2010 criteria. Semen samples were evaluated in terms of liquefaction and viscosity within 30–60 min after ejaculation. Viscos samples were liquefied using α-chymotrypsin then the semen volume was measured. Semen samples were microscopically evaluated in terms of aggregation, agglutination, epithelial cells, and round cells such as leukocytes and immature germ cells. Spermiogram analyses were performed using an SQA-V Gold spermiogram device.

### 2.3. Sperm smear preparation

Sperm smears were made for both sperm morphology and immunohistochemical analysis. The ejaculate was mixed one to five (1:5) ratio with phosphate buffer solution (PBS) in test tubes, then it was centrifuged and the supernatant was removed. After washing, a small amount of the pellet diluted with PBS was dropped on the slide, and sperm smear preparations were made. Slides were dried at room temperature and then fixed with 4% neutral buffered formalin for 10 min.

### 2.4. Diff-Quik staining

The Diff-Quik stain kit consists of fixative (methanol), solution-I (eosinophilic xanthene), and solution-II (basophilic thiazine) agents. The sperm smear slides were left sequentially for 10–15 s in each solution, followed by a water rinse and drying. The slides were mounted with a coverslip using Entellan for long-term use and analyses under a microscope. Kruger strict sperm morphologic analyses were performed on the stained preparations in accordance with the WHO-2010 criteria [18, 19].

### 2.5. Immunohistochemistry staining

An immunohistochemistry protocol was performed on the semen smears to detect immunohistochemical expressions and the immune location of AQP-3 (Thermo Fisher Scientific USA), AQP-7 (Protein tech USA), and AQP-8 (Abcam UK) molecules in sperm cells. In brief, the slides were washed in PBS and incubated for 15 min in 1% bovine serum albumin (BSA) for blocking. The BSA was removed without washing then primary antibodies of AQP-3 (1:100), AQP-7 (1:100), and AQP-8 (1:100) were dropped onto the samples and incubated at 4 °C in a humidified and dark chamber overnight. After washing with PBS for 5 min at room temperature, the samples were incubated with biotinylated secondary antibody (Vector Laboratories, USA) at room temperature in a humidified dark environment for 30 min and again washed with PBS incubated with horseradish peroxidase (HRP)-labeled streptavidin secondary antibody (GE Healthcare UK) at room temperature for 20 min. After washing with PBS, immune expressions were made visible adding diaminobenzidine (DAB) chromogen (Sigma, Germany) onto the samples. For the negative control, some slides were simultaneously treated with PBS instead of the primary antibodies. The immunochemical stained slides were mounted with a coverslip following counterstaining with hematoxylin and washing with distilled water.

### 2.6. Immunohistochemistry analysis

**Figure 1:**
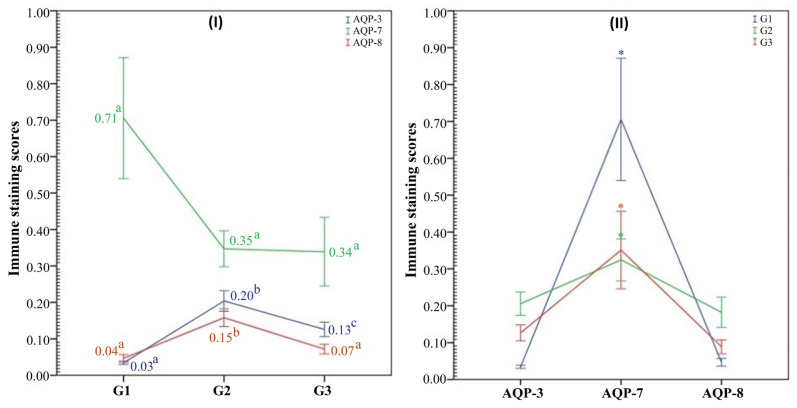
The first graph (I) shows the mean immune staining intensity scores of AQP-3, AQP-7, and AQP-8 molecules in each group. Different letters on mean values express statistical significance (p < 0.05). The second graph (II) demonstrates the comparison of each AQP molecule immune expression for the same group. Asterisks on the bar indicate statistical difference (p < 0.05).

The intensity of AQP-3, 7, and 8 immunostaining was evaluated using the Nikon NIS-Element software (Hasp ID: 6648AA61; Nikon) through a Nikon Eclipse E200 research microscope. In each slide, six fields were randomly selected and searched at ×1000 magnification to detect immune staining densities and locations in the sperm cells. To determine the expression levels of the AQP molecules, the immunostaining intensity of these molecules was graded at four categorical levels according to an immunostaining scoring scale [20]. The criteria in this scale are given in detail in Table 1. The mean immunostaining score of each participant was calculated, then the average immunostaining scores of the groups were measured and the data were compared statistically (Figure 1).

### 2.7. Statistical analysis

Statistical analyses were conducted using the Statistical Package for the Social Science (SPSS) for Windows, version 22 software (SPSS, IBM Co., Somers, NY, US). After testing the homogeneity of variances, a one-way analysis of variance (ANOVA) was used for statistical analysis, as appropriate. All data are shown as mean ± standard error mean (SEM). Kruger strict and spermiogram analyses results were compared using Tukey’s honestly significant difference (HSD) test, and immunohistochemistry analyses were compared using the Tamhane test. P-values of <0.05 were considered statistically significant.

## 3. Results

### 3.1. Spermiogram test results

The volume of semen, one of the macroscopic parameters of the spermiogram, decreased gradually in group 1, group 2, and group 3. Accordingly, it was observed that semen volume tended to decrease correlation with aging. No differences were found among the groups in terms of nonmicroscopic parameters such as liquefaction, viscosity, appearance, pH, and aggregation. The analyses of some of the spermiogram tests of the groups are shown in Table 2. Sperm concentration values were found to be the highest in group 3 and gradually decreased in groups 1 and 2, respectively. Motile sperm (progressive + nonprogressive sperm) concentrations of the groups were statistically similar (p > 0.05) (Table 2). Although some subtle differences were observed, no statistical difference was found in terms of the spermiogram test results of the groups.

### 3.2. Kruger strict sperm morphology analysis results

**Figure 2:**
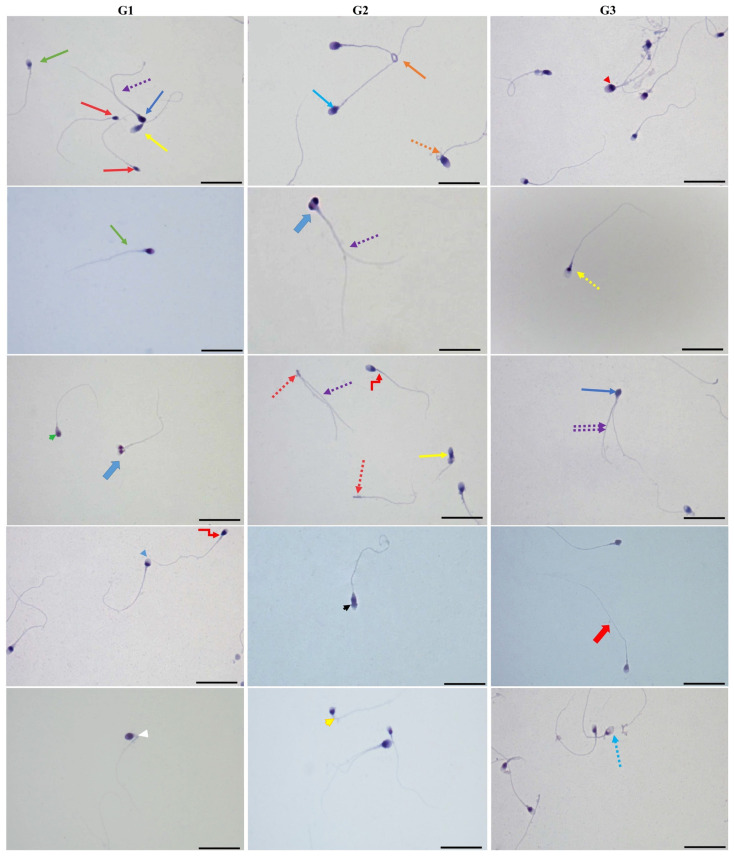
Representative microscopic examples of sperm anomalies are most seen in the Kruger strict analyses of the study groups. In the pictures; green arrow: normal, red arrow: small head, yellow arrow: elongated head, dark blue arrow: without acrosome, dotted purple arrow: double-tailed, double dotted purple arrow: three-tailed, blue arrow: vacuole acrosome, orange arrow: coiled tail, dotted orange arrow: cytoplasmic residues, red arrowhead: large-headed, blue arrowhead: round-headed, curved red arrow: nonaxial middle piece, dotted red arrow: headless, blue thick arrow: double head, dotted yellow arrow: excess acrosomes, green arrowhead: apical nucleus, black arrowhead: incomplete acrosome separation, yellow arrowhead: curved neck, white arrowhead: bent neck, thick red arrow: long-tailed sperm cells (Diff-Quik, Scale bars: 20 μm).

Kruger strict morphology analyses of Diff-Quik stained sperm cells were evaluated at the three main regions of the sperm cells, the head, neck, tail, and as a whole. We found that sperm head anomalies were more frequent than in other regions of sperm, but there were no statistical differences among the groups in terms of these anomalies (p > 0.05). In the overall evaluation of the anomalies, the numbers of sperm with anomalies were shown to slightly increase with age, but no statistical difference was found between the groups (p > 0.05) (Tables 2 and 3) (Figure 2).

### 3.3. Immunohistochemistry analysis results

**Figure 3:**
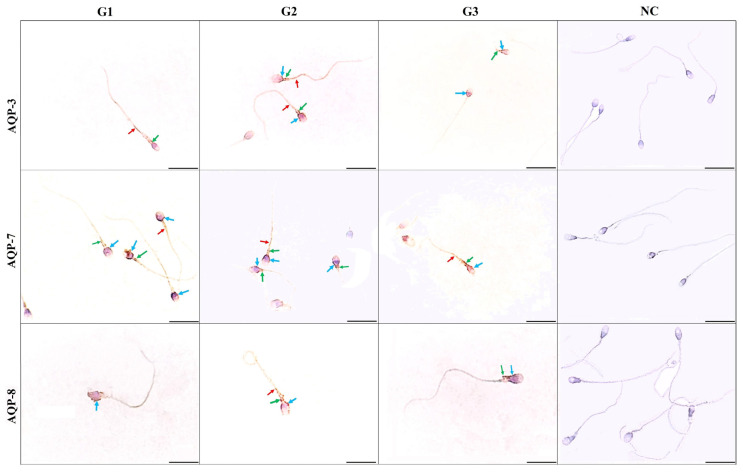
Representative microscopic images of the immune expression and location of aquaporin molecules in sperm cells from each study group (G1: group1, G2: group2, G3 group3, and NC: negative control immune images). On the sperm cells, blue, yellow, and red arrows indicate the head, neck, and tail of spermatozoa, respectively (IHC-DAB, Scale bars: 20 μm).

The mean immunostaining intensity scores and immune location of AQP molecules in the sperm cells of the study groups are shown graphically in Figure 1 and representative microscopy images in Figure 3. AQP-3 immune staining was mostly seen in the main part of the sperm tail, middle piece of the sperm, and also lightly stained in the sperm head and around the region. In normal sperm, AQP-7 immunostaining was mostly detected in sperm heads as dense staining (Figure 2), whereas in abnormal sperm, it was also seen in other regions. AQP-8 immunoexpression was a little stronger in the middle piece of abnormal sperm, especially in thick-necked sperm, whereas it was lightly stained in normal sperm. We compared the immunostaining scores as a whole because no statistically significant differences were detected in terms of the regional location of AQPs. The immunostaining score of AQP-3 was the lowest in group 1 and increased in group 3 and group 2, respectively (p < 0.05). AQP-7 immunostaining scores were not different between the groups (p > 0.05) (Figure 3, first graphic). AQP-8 immunostaining scores only increased in group 2 (p < 0.05). When the immunostaining scores of AQP molecules were compared with each other, it was determined that AQP-7 was significantly higher (p < 0.05) than the others (Figure 3, second graphic).

## 4. Discussion

Paternal infertility is directly related to the availability of many quality sperm cells in terms of basic spermiogram parameter criteria such as the motility, number, and morphology of semen. Apart from the known, many factors that have not yet been discovered may also play a crucial role in sperm quality and parameters. We considered that some AQP molecules seen in sperm cells, such as AQP-3, AQP-7, and AQP-8, could play a role in causing this situation. Accordingly, we aimed to investigate the relations of expression of AQP molecules in the sperm cells of different aged men. Therefore, we conducted experimental analyses such as immunohistochemistry, spermiogram, and Kruger strict morphology tests of sperm cells from three different age groups of human semen samples.

Semen volume, one of the macroscopic parameters of the spermiogram, tended to decrease with age. When evaluated in terms of other parameters such as liquefaction, viscosity, appearance, aggregation, and pH, there was no significant difference among the groups according to age. These and other spermiogram parameters such as concentration and total sperm count values were not significantly different from each other, as we expected. This may be because few patients were aged over 40 years.

In Kruger strict morphology analysis, the normal sperm percentage was above 4%, which was in the normal range. The percentage of sperm with normal morphology was the highest in group 1 and slightly decreased in groups 2 and 3. As expected, an increase in the total number of sperm anomalies was also observed in correlation with age. However, all these spermiogram and Kruger results were not statistically different between the groups; therefore, all these findings imply that the groups had a standardization for the investigation of AQPs using immunohistochemical expression.

Besides other factors such as oxidative stress, the hypotonic environment is also an important mechanism that can affect sperm morphology and motility. Studies have reported that AQPs have important roles in these mechanisms. In this context, it has been alleged that AQPs increase sperm cell survival and fertilization capacities [21–25].

It was suggested that AQP-3 played a role in the physiologic hypotonic stress required for sperm motility activation [26, 27] and regulatory volume decrease, which is common in the sperm of patients with infertility, inhibits the movement of sperm, and provides the balance between cell swelling and tail bending [28]. In animal studies, it has been reported that AQP-3 is also associated with sperm cell cryotolerance [23, 24].

In the study of Chen et al. (2011), no significant difference was found between normal males and AQP-3 knockout (AQP-3 −/−) males in terms of sperm morphology and number [28]. It was also found that AQP-3 was located in the membrane of the principal part of the sperm tail [28]. On the other hand, Laforenza et al. (2017) showed that AQP-3 molecules, in 3% of the sperm, were found in the sperm head and middle part, as well as in the main part of the sperm tail [29]. In our study, AQP-3 immune expression was mostly seen in the main part of the sperm tail, the middle piece of the sperm, but scarcely in the sperm head region. We observed that AQP-3 expression increased with age, although group 3 had slightly less compared with group 2. It may imply that AQP 3 molecules may have a more active role in these periods of life than in other periods.

In some studies, AQP-7 molecules were detected in the head region [29], middle piece, and proximal tail regions of sperm cells [30]. In other two different studies, it has been reported that increased AQP-7 characterization was correlated with progressive motility [30, 31]. Moretti et al., (2012) mostly detected AQP-7 in the pericentriolar and equatorial regions of normal sperms, but little in the tail region. In abnormal sperm, they also detected AQP-7 in cytoplasmic residues, coiled tails, head, and acrosome [31]. In another study, it was suggested that a male with AQP-7 knockout could be fertile [32]. It has been reported that AQP-7 is associated with the cryotolerance of sperm cells of wild boar and bull [24, 33], but not those of stallions [23]. Our study results show some similarities to those of Moretti et al. We encountered that in normal sperm, AQP-7 was mostly located in the sperm head with densely staining (Figure 2), whereas in abnormal sperm, it was also observed in other regions of sperm cells. Therefore, AQP-7 may be associated with sperm motility and activity, as stated in the studies of Yeung et al., and Saito et al. (2004). As we understood from all the studies, AQP-7 is located in all regions of both normal and abnormal sperms distinctly. It might be the reason why AQP-7 expressed more than the other AQPs.

AQP-8 molecules have been suggested to play a role in the reduction of cytoplasm during the differentiation of spermatids into spermatozoa [34]. AQP-7 and AQP-8 expressions through life were detected significantly higher in rats, between the 15th and 20th days after birth [35]. It has been reported that these molecules play an important role in the adaptation of sperm to osmolality changes [15]. Laforenza et al. (2017) detected AQP-8 around mitochondrion in the middle piece of spermatozoa in 2% of sperms [29]. In another study, AQP-8 expression was shown in sperm tails in a granular form [15].

A study conducted on human sperm reported that AQP-8 was located in the cytoplasm and tail membrane in a discrete pattern and thus was not associated with sperm motility. Although it was inversely proportional to bent sperm, no difference was observed between patients and donors [15]. In the present study, AQP-8 was scarcely seen in normal sperm, whereas there was slightly more in the middle piece of abnormal sperm, especially in thick-necked sperm.

Our study has some limitations: Semen samples were obtained from a total of sixty men who applied for infertility at two centers, and there were not too many participants. We used only spermiogram, Kruger, and immunohistochemical techniques and could not utilize other methods like western blotting.

There is a paucity of studies in the literature on the relationship between AQP molecules and male infertility, especially in human sperm cells. In these existing studies, we encountered inconsistent findings related to the location and characterization of AQP molecules. A considerable amount of new research is needed; therefore, we expect this study will be crucial and will expand the knowledge in this area.

As a result, we observed characterization of AQP-3 and AQP-8 at age 26 to 35 years, and those of AQP-7 molecules were greater at 18 to 25 years in human sperm cells. AQP-7 immunoexpression was slightly stronger than both AQP-3 and AQP-8 in all groups. The results imply that AQP-3 and AQP-8 have a more active role in the age of 26–35 than those in both early and late periods of fertility. While AQP-7 has a more active role in the beginning infertility period, later its action is slightly decreasing and stabilizing. AQP-7 is also more active than both AQP-3 and AQP-8 in human sperm cells. If the expression of these molecules is adversely affected, male infertility may eventually emerge. We recommend further advanced level studies on the effects of these molecules characterized in sperm cells on male infertility.

## Figures and Tables

**Table 1:** Criteria for immune staining scoring scale.

Score	Immune reactivity
0+	No staining
1+	Weak, but detectable staining
2+	Moderate staining
3+	Strong/intense staining

**Table 2:** SQA Spermiogram test mean values of each group.

	Sperm/mL	Kruger	Total sperm
Group-1	67.61 ± 13.3	16.52 ± 1.9	225.20 ± 45.9
Group-2	56.37 ± 7.9	14.10 ± 1.3	185.35 ± 27.5
Group-3	89.92 ± 13	14.79 ± 2.3	312.00 ± 51.9
p	>0.05	>0.05	>0.05

Data are expressed as mean ± SEM.

**Table 3:** Mean values of morphological analysis results (±: SEM).

	Sperm anomalies				
	Head	Neck	Tail	Total	Anomaly%
Group-1	142.3 ± 8.1	25.70 ± 4.9	22.00 ± 3.4	90.00 ± 7.0	83.20 ± 2.6
Group-2	139.6 ± 7.4	31.80 ± 6.5	32.10 ± 6.3	203.50 ± 7.9	87.37 ± 1.4
Group-3	152.5 ± 9.1	20.80 ± 3.3	30.50 ± 7.1	203.80 ± 10.0	87.37 ± 3.0
p	>0.05	>0.05	>0.05	>0.05	>0.05

Data are expressed as mean ± SEM.
